# The temporal increase in HIV-1 non-R5 tropism frequency among newly diagnosed patients from northern Poland is associated with clustered transmissions

**DOI:** 10.7448/IAS.18.1.19993

**Published:** 2015-08-20

**Authors:** Miłosz Parczewski, Magdalena Leszczyszyn-Pynka, Magdalena Witak-Jędra, Katarzyna Maciejewska, Sławomira Myślińska, Anna Urbańska

**Affiliations:** Department of Infectious, Tropical Diseases and Acquired Immune Deficiency, Pomeranian Medical University, Szczecin, Poland

**Keywords:** V3 sequencing, temporal trends, tropism, Bayesian inference, clustering

## Abstract

**Introduction:**

CCR5 (R5) tropic viruses are associated with early stages of infection, whereas CXCR4 (X4) HIV-1 tropism has been associated with severe immunodeficiency. We investigated the temporal changes in the genotype-predicted tropism frequency and the phylogenetic relationships between the R5 and non-R5 clades.

**Methods:**

A cohort of 194 patients with a newly diagnosed HIV infection that was linked to their care from 2007 to 2014 was analyzed. Baseline plasma samples were used to assess the HIV-1 genotypic tropism with triplicate V3-loop sequencing. The non-R5 tropism prediction thresholds were assigned using a false positive rate (FPR) of 10 and 5.75% and associated with clinical and laboratory data. The transmission clusters were analyzed using *pol* sequences with a maximum likelihood and Bayesian inference.

**Results:**

The overall non-R5 tropism frequency for 5.75% FPR was 15.5% (*n=*30) and 27.8% (*n=*54) for 10% FPR. The frequency of the non-R5 tropism that was predicted using 5.75% FPR increased significantly from 2007 (0%) to 2014 (*n=*5/17, 29.4%) (*p=*0.004, rough slope +3.73%/year) and from 0% (2007) to 35.3% (2014, *n=*6/17) (*p=*0.071, rough slope +2.9%/year) using 10% FPR. Increase in the asymptomatic diagnoses over time was noted (*p*=0.05, rough slope +3.53%/year) along with a tendency to increase the lymphocyte CD4 nadir (*p*=0.069). Thirty-two clusters were identified, and non-R5 tropic viruses were found for 26 (30.95%) sequences contained within 14 (43.8%) clusters. Non-R5 tropism was associated with subtype D variants (*p*=0.0001) and the presence of CCR5 Δ32/wt genotype (*p*=0.052).

**Conclusions:**

R5 tropism predominates among the treatment of naive individuals, but the increases in the frequency of non-R5 tropic variants may limit the clinical efficacy of the co-receptor inhibitors. The rising prevalence of non-R5 HIV-1 may indicate transmission of X4 clades.

## Introduction

HIV-1 entry into the target cell requires the use of the CCR5 or CXCR4 co-receptor [1]. Circulating HIV clades may exhibit tropism to one or both of the co-receptors and are classified as R5, X4, or dual/mixed (D/M) tropic for the CCR5, CXCR4, or D/M tropic variants, respectively [2]. As R5 tropic variants predominate in the primary and early stages of infection, previous reports suggested the possible preferential transmission of CCR5 utilizing variants, with the mucosal barrier acting as a factor driving this genetic bottleneck [3]. With the progression of HIV disease, the frequency of non-R5 clades increases and reaches approximately 50% [4]. X4 viruses have been associated with faster lymphocyte CD4 decline compared with the R5 clades, but may also reflect delayed HIV diagnosis [5].

With the introduction of CCR5 inhibitors in the clinical practice, sequence-based assays to predict HIV-1 tropism have been developed and validated [6]. Tropism predictions based on the sequencing of the third hypervariable (V3) loop allow clinicians to distinguish between R5 and non-R5 (X4 and D/M) clades using the amino acid charge of the V3 region and various prediction algorithms, of which geno2pheno is the most popular [7]. Genotypic tropism predictions allow clinicians to not only select patients that may be susceptible to CCR5 inhibitors but also to screen large cohorts to observe the clinical and genetic characteristics associated with tropism and to analyze the spread of R5 and X4 viruses [8]. We have recently identified an association between the chemokine receptor genetic variants and tropism, with the CX3CR1 rs3732378 A allele being associated with increased prevalence of R5 tropic clades in newly diagnosed individuals [9]. In this study, we aimed to investigate the temporal changes in tropism frequency in the same dataset supplemented with 2013 and 2014 data and analyze the phylogenetic relationships between the R5 and X4 tropic clades using matched reverse transcriptase and protease sequences to characterize transmission events and clustering.

## Methods

### Study group

For the study data, we analyzed samples from 194 newly diagnosed treatment-naïve patients with confirmed HIV infection linked to care at the Department of Infectious, Tropical Diseases and Acquired Immune Deficiency (Pomeranian Medical University, Szczecin, Poland) and Out Patients’ Clinic for Acquired Immunodeficiency (Regional Hospital, Szczecin, Poland) from 2008 to 2014. All patients reporting to the centre with the confirmation test within one year from the first visit date were included in the study. The study protocol was approved by the bioethical committee of Pomeranian Medical University, Szczecin, Poland (approval number KB-0012/08/12 for HIV-1 tropism and BN-001/34/04 for CCR5 Δ32 genotyping), and consent was obtained from the patients included in the study. Plasma samples collected prior to the introduction of antiretroviral treatment were used for HIV-1 tropism assessment. Whole blood samples were used for DNA extraction and CCR5 Δ32 genotyping.

The following clinical data were collected: age, sex, date of HIV diagnosis, route of transmission, hepatitis C co-infection, clinical category at diagnosis according to the CDC case definition, baseline HIV viral load, and the baseline and nadir lymphocyte CD4+ counts. The baseline lymphocyte CD4 counts were defined as the first documented result after HIV diagnosis.

The CDC category at diagnosis was assumed based on a review of the patient's clinical record. Polymerase chain reaction (PCR) with sequence-specific primers was used to analyze the CCR5 Δ32 (rs333) variation, according to the previously described PCR methodology [10, 11].

### HIV-1 tropism assessment

V3 loop sequencing was performed according to the methodology provided by the HIV Centre of Excellence (personal communication, prof. R. Harrigan) from the first collected plasma sample of the HIV-1 diagnosed treatment-naive patients. Briefly, nested PCR was performed after reverse transcription of the extracted HIV-1 RNA. The amplicons were used for sequencing by standard techniques using an ABI 3500 platform (Applied Biosystems, Foster City, CA). Analyses were performed in triplicate. Two overlapping sequencing reactions (forward and reverse) were performed for each sample. Standardized operating procedures were used to ensure lack of contamination within the sequences; RNA extraction, reverse transcription and amplification were performed in the separated dedicated laboratory rooms. The sequences were assembled using the Recall online tool (www.pssm.cfenet.ubc.ca), which also provided sequence-based HIV-1 tropism interpretations using the geno2pheno algorithm [12]. The same version of tropism interpretation algorithm was used for all the sequences included into the study. Non-R5 tropism prediction thresholds were assigned using a false positive rate (FPR) of 10% as defined by the European Guidelines on HIV-1 tropism testing [6] and 5.75% FPR as defined by the MERIT and MOTIVATE trials [13]. Across the triplicates, discordant tropism results were found in seven (3.6%) cases for FPR 10% and two (1%) for FPR 5.75%.

### Subtyping and phylogenetic analyses

Bayesian inference was used to analyze the phylogenetic relationships between the sequences with a similar predicted tropism. As the tropism sequences were short and triplicate testing confounds the phylogenetic analysis, we used the 1302 bp (HXB2 genome location 2253 to 3525) protease/reverse transcriptase sequences obtained by standard Viroseq 2.8 genotyping of the same samples. As one sequence was notably shorter (<1000 bp long), 193 sequences were included for the phylogenetic analyses. First, the sequences were aligned with Clustal X2.0.10 (www.clustal.org) software [14]. A GTR model with empirical nucleotide frequencies was selected with the jModeltest 2.1.1 software [15]. The nucleotide frequencies calculated under this model were as follows: freqA=0.4179, freqC=0.1598, freqG=0.1985, freqT=0.2238, gamma shape parameter=0.903 and p-inv=0.462.

To investigate the existence of clusters with similar tropism, the maximum likelihood (ML) method with the NNI-SPR sub-tree algorithm and the GTR model with the PHYML v 3.0 software online web server were used to compute evolutionary distances and calculate the aLRT values [16]. In addition, we used a Bayesian Monte Carlo Marcov Chain (MCMC) for the analyses. Two replicates of 100 million generations were run in BEAST v. 1.5.3 [17] using a constant population size and a GTR+Γ with uncorrelated lognormal relaxed molecular clock. All prior and posterior effective sample size values exceeded 200 [18]. Clustering was assessed using Cluster Picker software with the maximum genetic distances calculated by the program. Clusters were assigned with the aRLT value>90% for the ML method and a posterior value >95% for Bayesian inference; in both cases, maximum pair-wise distances <0.045 were used [19]. A consensus tree with posterior probabilities for branch support was obtained and annotated with TreeAnnotator v 1.5.4. All trees were visualized in Figtree v.1.2.2.

To evaluate number of recent infections in the sample fraction of ambiguous nucleotides in partial *pol* sequences was used; >0.5% ambiguity cutoff value suggestive of the recent infection was implemented, according to the previous study [20].

Subtyping was performed using genotyping software (REGA genotyping 2.0 tool; www.bioafrica.mrc.ac.za/rega-genotype/html/subtypinghiv.html) based on the partial *pol* sequences used in the study. Genbank reference sequence numbers used in this manuscript were as follows: GU906869, GU906871-GU906877, JQ305761-JQ305767, KM057350-KM057351, KM057353, KM057354, KM057357, KM057361, KM057362, KM284492-KM284623, KM284626-KM284660, KM284663, KM284664 and KM284667-KM284680.

### Statistical analyses

Fisher's exact and chi-square tests were used for the nominal variables, and U-Mann Whitney/ANOVA tests were used for the continuous variables (Statistica PL 8.0, Statasoft, Poland). Time trends were examined using logistic regression (R statistical platform, v. 3.1.0) for the binary variables and linear regression (Statistica PL 8.0, Statasoft, Poland) for the continuous variables. To validate the results, we have calculated the power of the sample sizes based on the assumption that the population size in the region for the years 2008 to 2014 was 500 cases (total number of newly diagnosed cases followed up in the centre increased by the coefficient of 30% (estimated percentage of undiagnosed HIV infections in Poland)). Based on the observed tropism frequencies, for the FPR 5.75%, the 95% CI sample size was 168 cases providing 4.57% margin of error, whereas for the FPR 10%, the 95% CI sample size was 191 cases, providing 4.94% margin of error.

## Results

### Group characteristics

The overall frequency of non-R5 tropism for the 5.75% FPR was 15.5% (*n*=30) and 27.8% (*n*=54) for the 10% FPR. The majority of patients in the study were male (135, 69.59%) and were infected by sexual contact (157, 80.92% which includes 79 (40.72%) heterosexually infected cases and 78 (40.21%) men having sex with men (MSM)) (Table 1). The symptomatic or AIDS stage of HIV infection was observed in most of the cases at care entry (102, 51.03%). The median CD4 lymphocyte count at care entry and nadir was <350 cells/µl, whereas the HIV-1 viral load exceeded 5 log copies/ml in 48.97% (*n*=95) of cases, including 12 (12.63%) for non-R5 5.75% FPR tropism and 22 (23.16%) for non-R5 10% FPR. Lymphocyte CD4 >500 cells/µl was observed in 10 (16.12%) and 18 (29.03%) for non-R5 5.75% FPR tropism and non-R5 10% FPR, respectively. Hepatitis C virus (HCV) coinfection was observed in 24.74% (*n*=48) of cases. In the analyzed dataset, 85 (43.81%) patients had ≤.5% ambiguous positions in their HIV sequence, 13 (43.33%) of 5.75% FPR-predicted non-R5 cases and 21 (38.89%) for 10% non-R5 FPR ones. No association was observed between the tropism and the ≤0.5% ambiguity score. As expected, HIV-1 subtype B was the most prevalent (148, 76.29%), followed by subtype D (27, 13.91%), A1 (8, 4.1%), C (3, 1.5%), CRF01_AE (3, 1.5%), CRF02_AG (2, 1%); and CRF13_cpx, CRF37_cpx and CRF28_BF (1, 0.5% each). There was no association between either the FPR 5.75% or FPR 10% predicted HIV tropism and age, sex, transmission route, infection stage, CD4 lymphocyte count at care entry and nadir, HIV-1 viral load, HCV coinfection, HIV-1 subtype, or CCR5 Δ32/wt genotype.

**Table 1:** Basic cohort characteristics

Variable (*n*=194)	Total
HIV-1 R5 tropism [5.75% FPR], *n* (%)	164 (84.53)
HIV-1 R5 tropism [10% FPR], *n* (%)	140 (72.16)
Male, *n* (%)	135 (69.59)
Age at care entry, median years (IQR)	36 (30–46)
HIV infection stage at genotyping, *n* (%)	
A	92 (47.42)
B	43 (22.16)
C	59 (30.41)
Dominant transmission route, *n* (%)	
HET (heterosexual)	79 (40.72)
MSM (men having sex with men)	78 (40.21)
IDU (intravenous drug use)	37 (19.07)
Lymphocyte CD4+ T cell counts at care entry, median cells/µl (IQR)	313 (113–559)
Lymphocyte CD4+ T cell counts at care entry <200 cells/µl, *n* (%)	80 (41.24)
Nadir lymphocyte CD4+ T cell counts, median cells/µl (IQR)	287 (104–512)
Nadir lymphocyte CD4+ T cell counts <200 cells/µl, *n* (%)	85 (43.81)
HIV-1 viral load at care entry, median log copies/ml (IQR)	4.97 (4.29–5.61)
Care entry HIV-1 viral load <5 log copies/ml, *n* (%)	99 (51.03)
HCV coinfected, *n* (%)	48 (24.74)
HIV-1 variant, *n* (%)	
B	148 (76.29)
Non-B variants	46 (23.71)
CCR5 Δ32/wt genotype frequency, *n* (%)	30 (15.46)
CCR5 Δ32/Δ32 genotype frequency	0

### Temporal trends

FPR increased significantly from 2007 (0%) to 2014 (*n*=5/17, 29.4%) (*p*=0.004, rough slope +3.73%/year) and from 0% (2007) to 35.3% (2014, *n*=6/17) (*p*=0.071, rough slope +2.9%/year) using 10% FPR.

**Figure 1:**
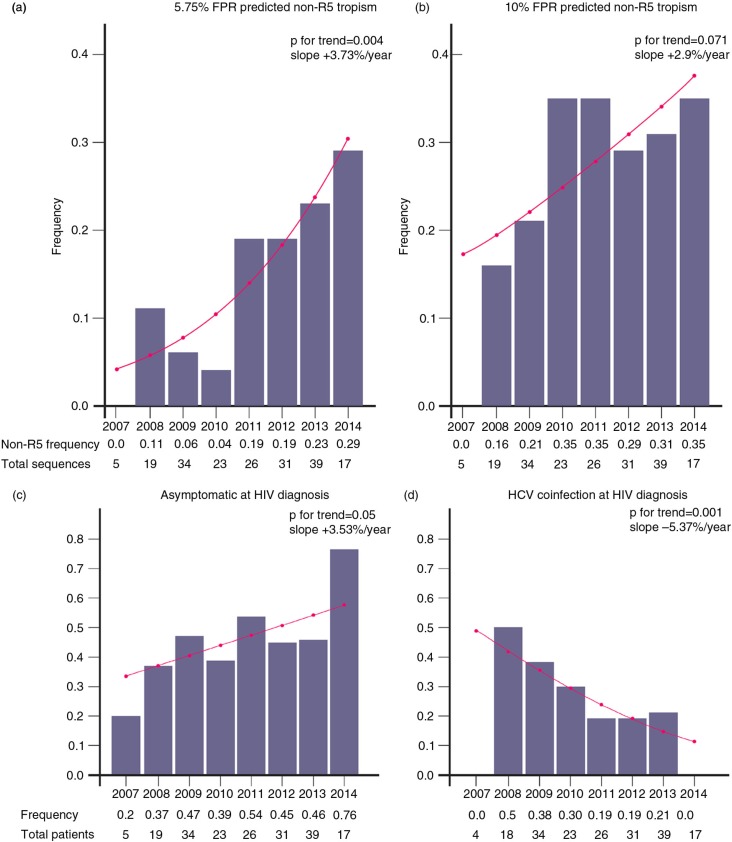
Proportions and logistic regression curves over time (2007–2014) for the non-R5 genotype-predicted HIV-1 tropism proportions with 5.75% (a) and 10% (b) false positive rates (FPR), asymptomatic HIV cases at the time of diagnosis (c) and HCV co-infections at the HIV diagnosis (d). Logistic regression curves are shown over the frequency bars.

The frequency of the non-R5 tropism predicted using 5.75% FPR increased significantly from 2007 (0%) to 2014 (29%, *n*=5/17) (OR:1.39 (95% CI:1.12–1.77), *p*=0.004, rough slope +3.73%/year) (Figure 1a). With 10% FPR, the frequency changed from 0% (2007) to 35% (2014 *n*=6/17) (OR:1.16 (95% CI:0.98–1.37), *p*=0.071, rough slope +2.9%/year) (Figure 1b). In addition, an increase in the asymptomatic diagnoses over time was noted (OR:1.15 (95% CI:1.0–1.34), *p*=0.05, rough slope +3.53%/year) (Figure 1c), but the frequency of AIDS at HIV diagnosis proved stable over time. A decreasing trend in the number of the intravenous drug use (IDU) compared to sexually (HET+MSM) acquired infections was observed (OR:1.16 (95% CI:0.97–1.4), *p*=0.1, rough slope +2.39%/year), but the frequency of heterosexual and MSM transmissions was stable over time. In accordance with this finding, there was significant decrease in the frequency of the HCV/HIV co-infections (OR: 0.75 (95% CI: 0.62–0.89), *p*=0.001, rough slope −5.37%/year) (Figure 1d). The temporal trends for sex distribution, non-B variant frequencies and CCR5 Δ32/wt genotype were not significant.

**Figure 2:**
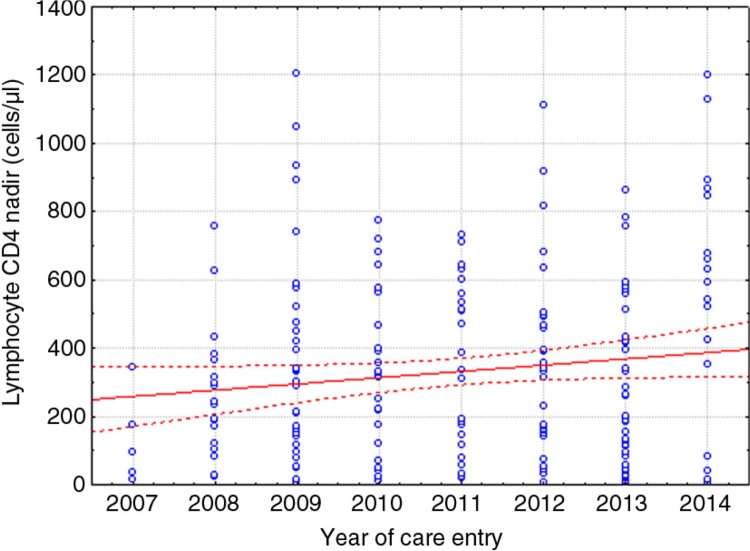
Linear regression temporal trend line (continuous curve) for the CD4^+^ lymphocyte nadir values from 2007 to 2014. The dotted lines represent the 95% confidence intervals, and the dots represent the raw CD4^+^ lymphocyte nadir values.

The pretreatment HIV-1 viral loads and CD4 lymphocyte counts at care entry were stable over time (*r*=(−0.012), *r* ^2^=0.0002, *p*=0.86 and *r*=0.07, *r* ^2^=0.0049, *p*=0.33, respectively); however, there was a trend towards increased CD4 lymphocytes at nadir over time (*r*=0.131, *r* ^2^=0.017, *p*=0.069) (Figure 2).

### Tropism clustering

**Figure 3:**
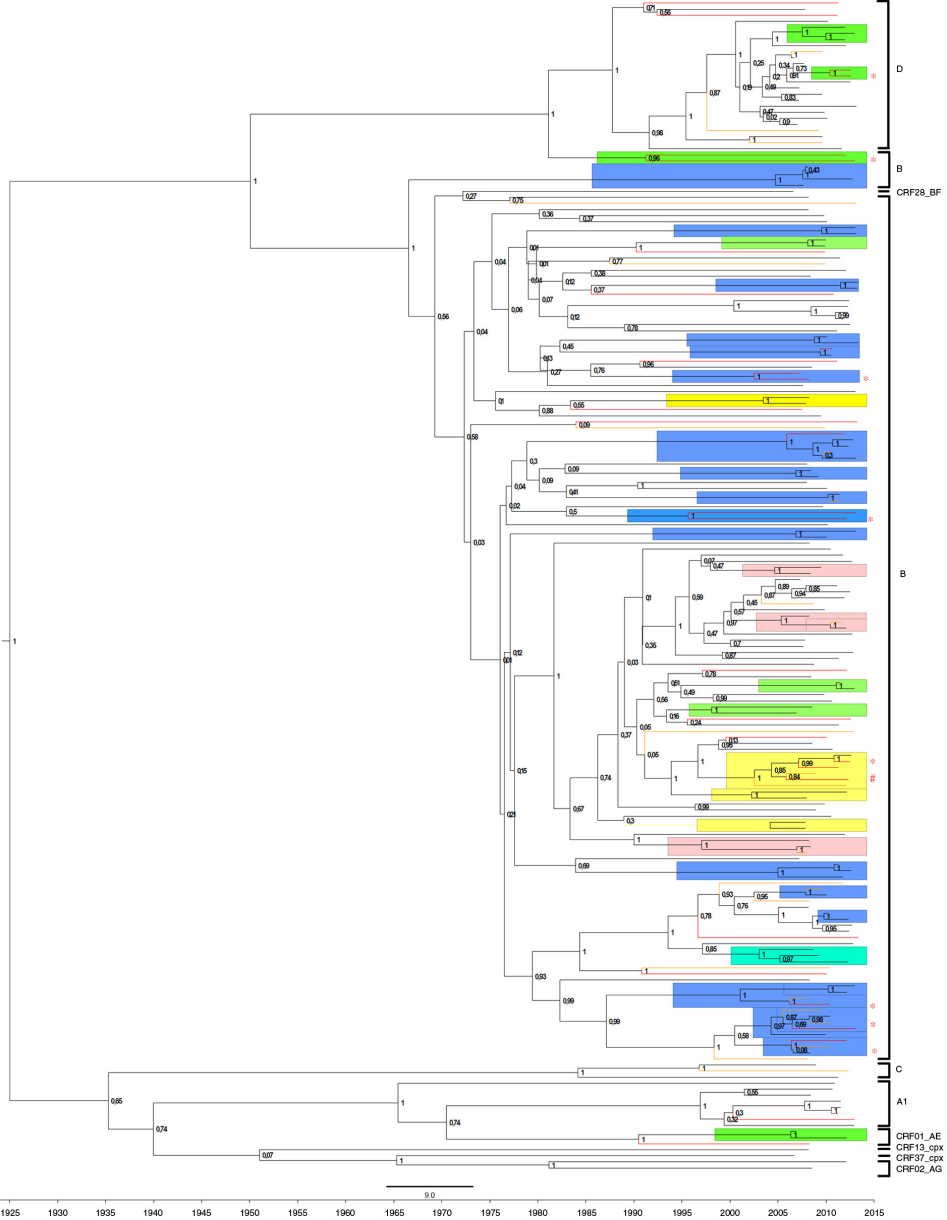
Bayesian, time-annotated MCMC phylogenetic tree of the subtype *pol* sequences corresponding to the tropism samples. The non-R5 tropism samples are indicated with red (FPR <5.75%) or yellow (FPR 5.75–10%). Transmission clusters identified by cluster picker with a maximum genetic distance <4.5%, a maximum likelihood value >90% and a Bayesian posterior >95% are highlighted and colour-coded: green for heterosexual, blue for MSM, pink for IDU and yellow for IDU/HET transmissions. The predominant clusters with possible circulation of non-R5 tropic viruses are marked with an asterisk. A mixed IDU/HET cluster with R5/non-R5 tropism is marked with #. Posterior probabilities exceeding 0.7 are shown on the tree nodes.

We constructed a phylogenetic tree containing 193 *pol* sequences corresponding to the samples used for the tropism assessment. The tropism as well as CCR5 Δ32 genotype were identified for every tip in the phylogenetic tree. In total, 32 clusters were identified and contained 84 (43.5%) sequences with 17 (53.1%) MSM transmission clusters, 7 (21.9%) heterosexual transmission clusters, 3 (9.4%) IDUs, 4 (12.5%) mixed heterosexual/IDU and 1 (3.1%) MSM/heterosexual cluster (Figure 3). It should be noted that 28 sequences were obtained from known partners and, therefore, most clusters containing only 2 isolates are pairs. Fourteen (43.8%) clusters contained 26 (30.95%) non-R5 tropic clades (FPR <10%). Of these, 4 clusters contained only non-R5 sequences, and both non-R5 and R5 tropic viruses were found in 10 clusters. A six-sequence cluster (marked with # on Figure 3) contained five non-R5 tropic clades (three injection drug users and two female sexual partners, may indicate a transmission network). The frequency of the pure non-R5 clades was more common within the clusters of the non-B (subtype D) variants (*p*=0.0001) and among patients with the CCR5 Δ32/wt genotype (*p*=0.052) (Table 2). The clusters with non-R5 sequences only were the most common among heterosexually infected cases compared to the MSM and IDU cases analyzed separately (*p*=0.008 and *p*=0.001, respectively) and to heterosexual vs. MSM vs. IDU (*p*=0.02).

**Table 2:** Characteristics of the sequence clusters based on V3 sequence-predicted tropism

	Genotype-predicted tropism for the analyzed sequences				
			
	Non-R5 only	Mixed non-R5/R5	R5 only	*p*	Total
Number of clusters (%)	4 (12.5)	10 (31.3)	18 (56.3)		32
Number of sequences (%)	8 (9.5)	35 (41.7)	41 (48.8)		84
Number of non-R5 clades (%)	8 (30.8)	18 (69.2)	0		26
Mean genetic distance within clusters, % (SD)	3.49 (0.16)	2.59 (1.06)	2.28 (1.2)	0.43^a^	
CCR5 Δ32 genotype frequency, number of patients (%)					
Δ32/wt	3 (25.0)	2 (16.7)	7 (58.3)	0.052^b^	12
Wt/wt	5 (6.9)	33 (45.8)	34 (47.2)		72
HIV variant, number of sequences (%)					
Subtype B	4 (5.3)	35 (46.7)	36 (48)	0.0001^b^	75
Non-B variants	4 (44.4)	0	5 (55.6)		9
Transmission mode, number of patients (%)					
HET	4 (19)	2 (9.5)	15 (71.4)	0.02^b^	21
MSM	4 (8.3)	23 (47.9)	21 (43.8)		48
IDU	0	10 (66.6)	5 (33.3)		15

The clusters were based on the maximum likelihood estimated distances from the corresponding partial *pol* sequences and assigned by Cluster Picker software with a ≥90% aLRT value, a 4.5% maximum genetic distance and verified using Bayesian inference in BEAST with a posterior probability ≥95%.

a ANOVA test;

b Fisher's exact test, two-tailed; HET – heterosexual transmission; MSM – men having sex with men; IDU – intravenous drug use.

## Discussion

The development of V3 sequence-based prediction algorithms for genotypic tropism assessment allowed us to not only test prior to CCR5 inhibitor introduction but also to investigate the influence of tropism on the clinical characteristics of HIV-positive patients [21–23]. It has been shown that the presence of X4/dual mixed viruses is associated with a more rapid progression of the infection and CD4 lymphocyte loss [5]. Transmission of the X4 variants was rare in recent seroconverters [3, 5], and it was suggested that R5 viruses are preferentially transmitted in both sexual and parenteral infections [1, 24]. However, the data on the existence of a mucosal bottleneck that limits the propagation of X4 viruses are contradictory. Linked transmissions of the X4 viruses were found among recently diagnosed HIV patients, which suggested random R5 and X4 variant transmission [8]. However, this finding was contrasted by data from Frang *et al*., who showed that clustered X4 variant transmission was not observed among primary HIV-1-infected individuals [25]. Overall, the frequency of the non-R5 tropism differs across cohorts and ranges from 1.4 to 19% for primary infections [3, 25, 26] to 9.1–38% [4, 8] in newly diagnosed cases, which is in accordance with the 27.8% (FPR10%) frequency of non-R5 variants identified in our study. Published studies indicate that increased non-R5 tropism frequency observed across the cohorts were associated with the higher number of late diagnosed patients and longer duration of the infection, lower baseline lymphocyte CD4 count and HIV subtype [3, 4, 8, 25, 26]. In our study, we found an increasing temporal trend for the frequency of the non-R5 tropism among newly diagnosed, treatment-naïve patients and clustered non-R5 variants. This trend is not likely to be associated with the delayed HIV diagnosis, as the number of patients with asymptomatic infection at diagnosis increased over time, and increases in the CD4 count at nadir and the number of AIDS cases were stable. Phylogenetic reconstructions indicate the possibility of the circulation of the non-R5 tropic viruses with clusters observed across all exposure groups, but they were most common among heterosexually infected cases. Moreover, the possibility of the sexual spread of the non-R5 variants is important in light of the decline of injection drug use-related transmissions. These findings are in accordance with the data presented by Chalmet *et al*., who found onward transmissions of X4 or D/M tropic viruses and the presence of X4 tropic viruses in 11% of the transmission clusters [8]. Furthermore, the spread of non-R5 tropic viruses was confirmed in 31% of well-characterized transmission pairs. This study allowed for the development of the “random transmission hypothesis,” which challenges the belief that R5 tropic viruses are selected during HIV-1 transmission [27]. Our study confirms the stochasticity of tropism spread, which results in the increase in the circulation of non-R5 tropic viruses and argues against the genetic bottleneck hypothesis and preferential infections with CCR5 utilizing variants. The temporal increase in the circulation of X4 tropic viruses, from 11.5% before 2001 to 23.3% in 2010 to 2012, was also found by Sierra-Enguita *et al*. in the cohort of the recent seroconverters from Spain [28]. As a majority of infections were related to the MSM contact, the sexual spread of X4 tropic viruses may be similar to that observed in our study.

Identification of the increased proportion of the non-R5 tropic viruses may also be related to the high HIV genetic diversity in our cohort and the duration of infection. Previously, a higher frequency of genotype-predicted X4 tropism was found in subtype D and CRF01_AE infections [8, 29]. This is in accordance with the data from our study, with more common non-R5 sequence clusters among the cases infected with subtype D. In fact, two sequence pairs from heterosexually infected cases contained V3 sequences with FPR <5%, indicating a possible transmission of subtype D non-R5 viruses. However, it is also possible that these heterosexually infected cases represent late testers with a higher likelihood of the R5 to non-R5 tropism switch. In Poland, there is only limited data on the viral relationship and frequency of clustering. Recently, in the multicentre sample of 833 antiretroviral treatment-naive individuals, we have identified that 20.9% of the subtype B sequences are transmission pairs and 23.7% are clusters ≥3 sequences, which indicates that the frequency of sequence clustering is similar across the country [30].

The higher frequency of the CCR5 Δ32/wt genotype in pure non-R5 clusters may reflect a loss of the beneficial effect in cases of exposure to X4/dual mixed tropic HIV-1. In the study by Brumme *et al*., individuals with CCR5 Δ32/wt heterozygote are at a significantly (2.5 times) higher risk of harbouring of the X4 variants [31]. It must be noted, however, that a preferential selection of the X4 variants in CCR5 Δ32/wt cases is also possible; to date, there is no conclusive data on whether the presence of the Δ32 allele is associated with lower susceptibility to R5 infections or favours a switch to CXCR4 using clades.

Our study has the following limitations: first, the recently diagnosed HIV-1 positive patients were analyzed, but the data on the duration of infection were not available. It was also impossible to distinguish between the acute and chronic HIV infections. Our analysis suggests the clustered transmission of the non-R5 variants; however, data from recent seroconverters supplemented with donor sequences would have to be used to exclude the possibility of the tropism evolution. A country-wide study would strengthen the conclusion on the possibility of the spread of the non-R5 tropic viruses in Central Europe. Second, there is only limited data for genotypic tropism assessments in non-B variants with the possible misinterpretation of genotyping tropism especially in the subtype D [29]. It was previously shown that subtype D specificity for non-R5 predictions was lower in comparison to subtype B [32]. Therefore, the tropism findings should be interpreted with caution [21, 33].

In conclusion, we found that the R5 tropism predominates among the treatment-naive individuals, but the increase in the frequency of non-R5 tropic variants may limit the clinical efficacy of the co-receptor inhibitors in the analyzed local population, which should be confirmed throughout the country. Increased prevalence of non-R5 HIV-1 may be related to the transmission of X4 non clades; however, this conclusion should be interpreted with caution. Circulation of the non-R5 tropic variants among treatment-naive patients is not only important for the loss of susceptibility to maraviroc but also for the possibility of the spread of the more rapidly progressing viral strains.
